# Kinetochore size correlates with chromosome size in Star of Bethlehem (*Ornithogalum kochii* Parl., Asparagaceae)

**DOI:** 10.1111/plb.70215

**Published:** 2026-04-14

**Authors:** K. Panda, M. Hroneš, F. Zedek

**Affiliations:** ^1^ Department of Botany and Zoology, Faculty of Science Masaryk University Brno Czech Republic; ^2^ Department of Botany, Faculty of Science Palacký University Olomouc Czech Republic

**Keywords:** Asparagaceae, centromere drive, chromosome size, kinetochore, KNL1, *Ornithogalum umbellatum* complex, recombination rate

## Abstract

- Chromosome segregation during cell division relies on the kinetochore, a multiprotein complex that mediates attachment of chromosomes to spindle microtubules. Biophysical considerations predict that kinetochore size should scale with chromosome size to meet increasing mechanical demands during segregation, yet empirical evidence for such scaling within karyotypes remains limited, particularly in species with modest chromosome size variation.
- Here, we investigated the relationship between chromosome size and kinetochore size in Star of Bethlehem (*Ornithogalum kochii*, Asparagaceae), a species from a genus with moderate intra‐karyotype chromosome size variation (2.4–4.5‐fold). Using immunolabelling of the conserved outer kinetochore protein KNL1 and linear mixed‐effects modelling, we analysed 172 chromosomes across multiple metaphases, slides and individuals.
- We detected a strong positive scaling relationship between chromosome size and kinetochore size, demonstrating that cytogenetic approaches can resolve chromosome–kinetochore scaling even across relatively narrow size gradients.
- Our results demonstrate that kinetochore size scales with chromosome size even in species with modest intra‐karyotype chromosome size variation, supporting the generality of chromosome–kinetochore scaling. Together with evidence from taxa exhibiting more extreme karyotype heterogeneity, this pattern suggests that centromere and kinetochore architecture is shaped by both functional constraints on chromosome segregation and long‐term evolutionary processes such as centromere drive. Viewed in a broader context, chromosome–kinetochore scaling may also be relevant for processes beyond segregation, as evolutionary changes in centromere and kinetochore architecture could indirectly influence recombination rates through their effects on chromosome size.

## Graphical abstract

Cytogenetic quantification of KNL1‐labelled kinetochores in *Ornithogalum kochii* reveals a significant positive scaling relationship between chromosome size and kinetochore size, demonstrating that chromosome–kinetochore scaling operates even across moderate intra‐karyotype size variation.

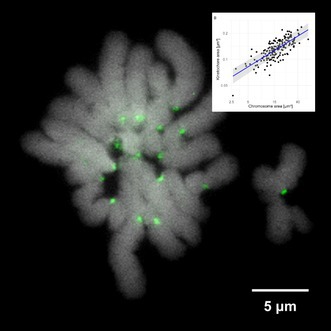

## INTRODUCTION

Chromosome segregation during mitosis and meiosis is mediated by the kinetochore, a large multiprotein complex assembled on centromeric chromatin that connects chromosomes to spindle microtubules. The kinetochore ensures both mechanical coupling to spindle microtubules and checkpoint surveillance, thus preventing chromosome mis‐segregation and aneuploidy (Foley & Kapoor 2013; Dou *et al*. 2019). Across eukaryotes, kinetochore architecture is broadly conserved (Wang *et al*. 2008; Xie *et al*. 2024). The core kinetochore protein is centromeric histone H3 (CENH3 or CENP‐A), which initiates the assembly of the inner constitutive centromere‐associated network (CCAN) and the outer network (KMN). KMN is further composed of kinetochore scaffold 1 (KNL1) complex, minichromosome instability 12 (MIS12) complex and nuclear division cycle 80 (NDC80) complex (Pesenti *et al*. 2016; Yatskevich *et al*. 2023; Xie *et al*. 2024).

Kinetochore size, typically approximated by the physical extent of kinetochore proteins, has important functional implications. Larger kinetochores can bind more microtubules (McEwen *et al*. 1998; Drpic *et al*. 2018), potentially providing greater robustness in chromosome–spindle interactions. In addition to the core kinetochore domain, microtubule attachment sites may also extend into pericentromeric regions in monocentric chromosomes or along kinetochore grooves in holocentric chromosomes, further contributing to attachment stability (Wanner *et al*. 2015). Conversely, both abnormally small and abnormally large kinetochores may increase the risk of erroneous attachments, leading to lagging chromosomes (Gregan *et al*. 2011; Drpic *et al*. 2018). Understanding how kinetochore size is determined and how it scales with chromosome features is therefore essential for elucidating the mechanisms of chromosome segregation and their evolution. During the movement in the viscous cytoplasmic environment, larger chromosomes experience greater drag forces and require more microtubule attachment sites to ensure timely segregation (Nicklas 1965; Kramer *et al*. 2021). Biophysical models of chromosome mechanics predict that kinetochore dimensions may be constrained by chromosome geometry (Kramer *et al*. 2021). Based on these considerations, we hypothesized that kinetochore size should scale with chromosome size (Plačková *et al*. 2021, 2022), reflecting both mechanical demands and general intracellular scaling principles (Levy & Heald 2012).

Across species, a relationship between total centromere size and genome size has indeed been documented in angiosperms (Bennett *et al*. 1981; Zhang & Dawe 2012; Plačková *et al*. 2021; Wang *et al*. 2021; Huang *et al*. 2026). Although this interspecific correlation is consistent with an underlying scaling relationship, it does not automatically imply that individual chromosomes within a karyotype follow the same pattern. Empirical studies addressing intra‐karyotype scaling are surprisingly few. Existing evidence from mammals, some grasses, *Arabidopsis* and algae suggests moderate correlations between kinetochore size and chromosome size (Jenkins & Bennett 1981; Cherry *et al*. 1989; Koornneef *et al*. 2003; Irvine *et al*. 2004; Wang *et al*. 2021, 2024; Huang *et al*. 2026). Our recent detailed study in the Asparagaceae subfamily Agavoideae, which possesses bimodal karyotypes with extensive variation in chromosome sizes, revealed a robust positive scaling relationship between chromosome size and kinetochore size (Plačková *et al*. 2022). However, it remains unknown whether similar scaling occurs in species with less extreme chromosome size variation.

The genus *Ornithogalum* L. (Star of Bethlehem, Asparagaceae) represents a suitable system for expanding this inquiry. *Ornithogalum* subgen. *Ornithogalum* exhibits intra‐karyotype chromosome size variation reaching ca. 2.4–4.5‐fold (Cullen & Ratter 1967; Tornadore & Garbari 1979; van Raamsdonk 1986; Dalgic & Özhatay 1997). Although not as extreme as in Agavoideae (Plačková *et al*. 2022), this variation should still be sufficient for quantitative analysis. Moreover, reliable kinetochore detection in monocots can be achieved using KNL1. This conserved outer kinetochore protein is involved in chromosome segregation and spindle assembly checkpoint signalling with lineage‐specific molecular features (Su *et al*. 2021; Deng *et al*. 2024) and has recently been validated as a robust marker of active kinetochore regions in vascular plants (Oliveira *et al*. 2024).

In this study, we examine whether the positive scaling relationship between kinetochore and chromosome size is also present in Star of Bethlehem. We used fluorescent immunolabelling of KNL1 to quantify functional outer kinetochore regions. Demonstrating such a pattern would support the hypothesis that chromosome–kinetochore scaling represents a general biological principle.

## MATERIALS AND METHODS

### Plant material

Bulbs of *Ornithogalum kochii* Parl. (2n = 18) were acquired from two natural populations in Czechia and Slovakia (Table S1). Plants were cultivated under controlled phytotron conditions (12 h light:12 h dark; 18 °C:13 °C) at the Department of Botany and Zoology, Masaryk University, Czech Republic. Root tips were collected from bulbs, and actively dividing root meristems were used for chromosome preparations.

### Chromosome preparation and immunolabelling

For chromosome preparation and immunostaining, we followed a previously published procedure (Plačková *et al*. 2022). Briefly, root tips were pre‐treated in ice‐cold water for 27 h, fixed in ice‐cold 4% formaldehyde solution in 1 × PBS (pH 7.4) for 25 min, washed and digested enzymatically in 1% cellulase R‐10 (Duchefa Biochemie), 1% pectolyase Y23 (Duchefa Biochemie) and 20% pectinase from *Aspergillus niger* (Sigma) at 37 °C for 1.5 h. After squashing the digested meristems, slides were frozen in liquid nitrogen. Slides were incubated with blocking solution (4% BSA, 0.01% Tween‐20, 1 × PBS) at room temperature for 1 h. After that, slides were incubated overnight at 4 °C with primary anti‐KNL1 antibodies (1:500). Purified polyclonal rabbit IgG antibodies recognizing a peptide corresponding to KNL1 (Neumann *et al*. 2023) were generated by LifeTein (USA). After washing in 1 × PBS, slides were incubated with Alexa Fluor 488‐conjugated anti‐rabbit secondary antibodies (1:500) at 37 °C for 1 h. Finally, slides were washed in 1 × PBS, dehydrated through a graded ethanol series (70%, 85% and absolute), and chromosomes were counterstained with DAPI (1.5 μg ml^−1^) in Vectashield (Vector Laboratories).

### Microscopy and measurement

Following Plačková *et al*. (2022), we used a simpler and less time‐consuming 2D approach for chromosome and kinetochore size analysis, as 2D and 3D measurements are strongly correlated. Images were acquired with an Olympus BX61 fluorescence microscope equipped with a ColorView II camera (Olympus) and Cell^F software. Chromosome and kinetochore areas were measured in wide‐field images using ImageJ v1.54d (Schneider *et al*. 2012). Only well‐separated chromosomes were analysed (for practical example see Fig. S1). The boundaries of DAPI‐stained chromosomes were determined with the ‘Freehand selection’ tool, while KNL1‐labelled kinetochore boundaries were identified using the ‘Elliptical selections’ function. Each kinetochore area corresponds to the average value of two sister kinetochores.

### Statistical analysis

To test the relationship between kinetochore and chromosome size, we applied linear mixed‐effects models implemented in the *nlme* package (Pinheiro *et al*. 2023) in R v4.3.1 (R Core Team 2023). The kinetochore area was set as the response variable and chromosome area as the predictor. To control for random variation among different metaphases, slides and plants, we treated these factors as nested random effects. Both variables were log_10_‐transformed before analysis to improve homoscedasticity and the normality of residuals. Partial residuals and the fitted relationship between chromosome and kinetochore size, accounting for random effects, were obtained using the *visreg* package (Breheny & Burchett 2017). Data were visualized using the *ggplot2* package (Wickham 2016).

## RESULTS

**Fig. 1:**
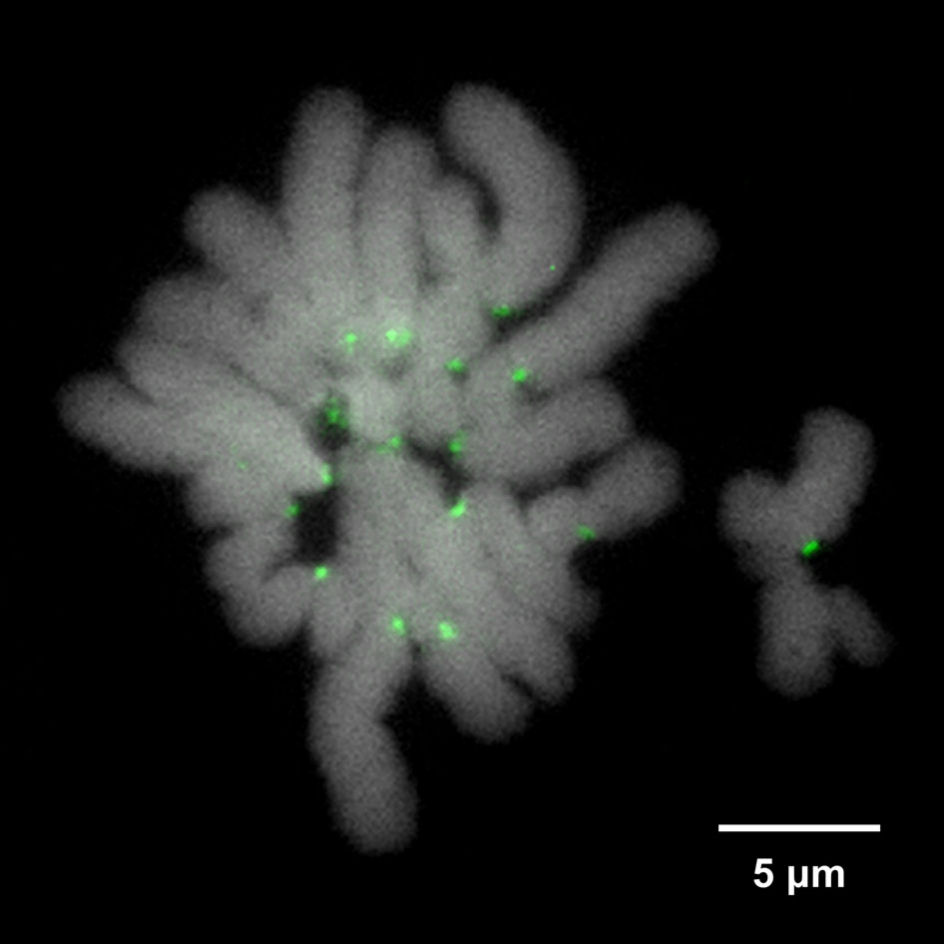
Immunolabelling of KNL1 (green) in the chromosomes of *Ornithogalum kochii*. Chromosomes were counterstained with DAPI (grey).

We quantified chromosome size using DAPI‐stained chromosome areas and estimated kinetochore size based on the spatial extent of the KNL1 immunosignal, which marks active outer kinetochore regions. KNL1 labelling was consistently detected at the centromeric regions of all analysed chromosomes, enabling reliable identification and measurement of individual kinetochores (Fig. 1).

In total, we analysed 172 chromosomes and their kinetochores across multiple metaphases, microscope slides and individual plants (Table S2). To account for the hierarchical structure of the data, we analysed the relationship between chromosome size and kinetochore size using a linear mixed‐effects model, with chromosome size included as a fixed effect and metaphases nested within slides and individual plants as random effects.

**Fig. 2:**
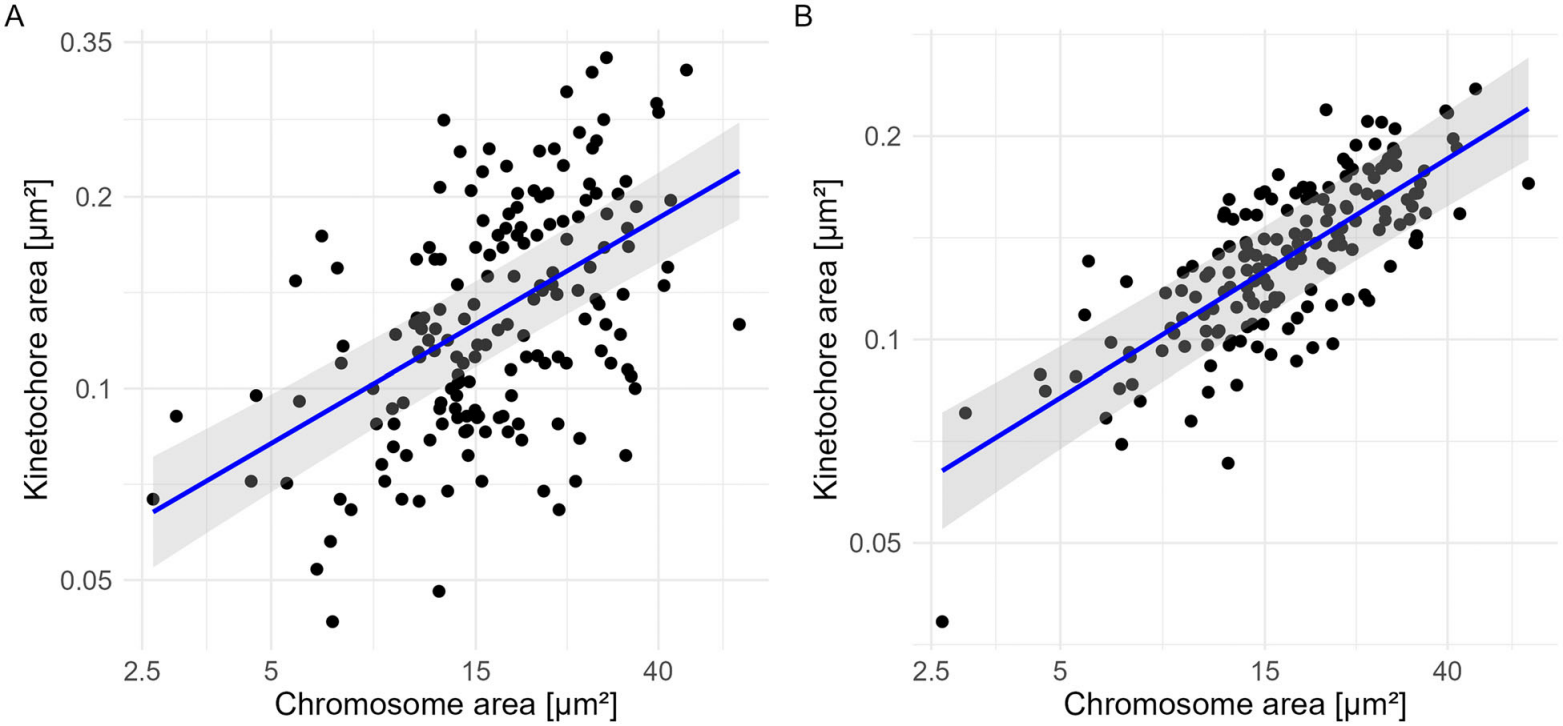
Scaling relationship of kinetochore area and chromosome area in Star of Bethlehem on a log_10_ scale. (A) The original data, including noise from the hierarchical data structure (metaphases within slides and individuals). (B) Partial regression plot showing the adjusted effect of chromosome size on kinetochore size after accounting for the noise caused by differences between metaphases, slides and individuals. The equation of both regression lines is y = 0.392x – 1.360 (n = 172).

The model revealed a strong positive relationship between chromosome size and kinetochore size, with an estimated slope of 0.392 that was highly significant (*P* < 0.001; Fig. 2 and Table 1). Chromosome size explained 24.86% of the variance in kinetochore size when considering fixed effects only (marginal R^2^). When both fixed and random effects were considered, the same model explained 78.33% of the total variance (conditional R^2^), indicating substantial variation associated with differences among metaphases, slides and individual plants. The substantial contribution of random effects likely reflects both biological and technical sources of variance, including differences among individuals, variation in root tip pre‐treatment timing, chromosome orientation inherent to 2D measurements, and possible differences in metaphase tension influencing the spatial extent of outer kinetochore components such as KNL1.

**Table 1:** Outcome of the regression model showing the effect of chromosome size on kinetochore size.

model term	b_i_	95% CI	*t*	*P*	R^2^ *mar*	R^2^ *con*
Intercept	−1.360	−1.414, −1.306	−25.042	0	24.86%	78.33%
Chromosome size	0.392	0.358, 0.426	11.572	0		

b_i_, parameters estimates; 95% CI, 95% confidence interval of the parameter estimate; *t*, *t*‐statistic; *P*, significance of the estimate difference from zero; R^2^ *mar*, marginal R‐squared indicating the proportion of variance in kinetochore size explained by the chromosome size; R^2^ *con*, conditional R‐squared indicating the proportion of variance in kinetochore size by the full linear mixed‐effects model (after accounting for random effects).

Despite this pronounced hierarchical variation, chromosome size remained a strong and highly significant predictor of kinetochore size. The positive scaling relationship was evident both in the raw data, which include variation among metaphases and experimental units (Fig. 2A), and in the partial regression plot illustrating the adjusted effect of chromosome size after controlling for random effects (Fig. 2B).

## DISCUSSION

Precise chromosome segregation requires stable attachment of chromosomes to spindle microtubules. Larger chromosomes are expected to experience greater mechanical demands during cell division, which may be fulfilled by an expansion of the kinetochore region, enabling the attachment of a larger number of spindle microtubules (Nicklas 1965; McEwen *et al*. 1998; Drpic *et al*. 2018; Kramer *et al*. 2021). In taxa with extreme intra‐karyotype chromosome size variation, these functional requirements are therefore expected to translate into pronounced differences in kinetochore size. This is exemplified by the subfamily Agavoideae, where chromosome sizes within a single karyotype may differ by up to an order of magnitude (~10‐fold; Watkins 1936; Kaneko 1966) and where a strong positive relationship between chromosome size and kinetochore size was previously demonstrated using a cytogenetic approach (Plačková *et al*. 2022). A similar relationship has been detected cytogenetically in humans, where chromosome sizes differ by approximately fivefold (Irvine *et al*. 2004).

In contrast, Star of Bethlehem exhibits comparatively modest intra‐karyotype chromosome size variation (Cullen & Ratter 1967; Tornadore & Garbari 1979; van Raamsdonk 1986; Dalgic & Özhatay 1997). Despite this relatively narrow size range, we detected a clear positive association between chromosome size and kinetochore size using a cytogenetic approach (Fig. 2 and Table 1). This result demonstrates that cytogenetic analyses are capable of resolving chromosome–kinetochore scaling even at moderate levels of intra‐karyotype size variation. At even finer scales of chromosome size variation, similar scaling relationships have been detected using genomic approaches. In maize, where the size difference between the largest and smallest chromosomes is approximately twofold, centromere and kinetochore scaling has been revealed through comparative genomic and ChIP‐based analyses (Wang *et al*. 2021). Furthermore, in species with even smaller differences in chromosome size, such as *Oryza sativa* and *Brachypodium distachyon*, Huang *et al*. (2026) reported a consistent trend towards positive chromosome–centromere scaling, although these relationships were not always statistically significant (Huang *et al*. 2026). Together, these findings indicate that chromosome–kinetochore scaling operates across a broad continuum of chromosome size variation, with the detectability of the relationship depending on both the magnitude of size differences and the resolution of the methodological approach.

The estimated slope of the scaling relationship (β = 0.392) exceeds the theoretical expectation (β = 0.25) derived from biophysical models of chromosome geometry and movement (Kramer *et al*. 2021), although it remains close to the range of empirically observed values reported across diverse taxa (β = 0.132–0.334; Irvine *et al*. 2004; Wang *et al*. 2021; Plačková *et al*. 2022). Deviations from the theoretical value may reflect both biological and methodological factors influencing kinetochore size estimation. In this study, kinetochore size was quantified using KNL1, a component of the outer KMN network that directly interfaces with spindle microtubules and whose spatial extent is known to respond to microtubule attachment and mechanical tension (Pesenti *et al*. 2016; Drpic *et al*. 2018; Yatskevich *et al*. 2023). Because the outer kinetochore exhibits greater structural plasticity than inner centromeric chromatin, KNL1‐based measurements may capture functionally relevant variation associated with chromosome segregation more directly than measurements based on CENH3‐marked centromeres (Irvine *et al*. 2004; Wang *et al*. 2021; Plačková *et al*. 2022). These biological and methodological sources of variability may also contribute to differences among metaphases, slides and individuals detected in the mixed‐effects model, and thereby to the observed difference between marginal and conditional R^2^ values (Table 1).

Beyond its role in mitotic chromosome segregation, chromosome size is tightly linked to meiotic recombination, with smaller chromosomes generally exhibiting higher recombination rates per unit length than larger ones across a wide range of taxa (Kaback *et al*. 1992; Jensen‐Seaman *et al*. 2004; Lynch *et al*. 2011; Näsvall *et al*. 2023; Zedek *et al*. 2026). This negative relationship implies that changes in chromosome size can affect recombination. In this context, the positive scaling relationship between chromosome size and kinetochore size is consistent with a mechanistic link between centromere/kinetochore architecture, chromosome size evolution and recombination rate. Centromere and kinetochore size can act as evolutionary constraints on chromosome size, as larger kinetochores permit the stable segregation of larger chromosomes, whereas smaller kinetochores limit chromosome expansion (Plačková *et al*. 2022, 2024). Consequently, any evolutionary process that modifies centromere or kinetochore architecture has the potential to alter chromosome sizes and thereby affect recombination rate variation over longer evolutionary timescales. In this broader evolutionary context, such constraints may be shaped by centromere drive, a rapidly evolving process in which centromeres compete for preferential transmission during asymmetric female meiosis (Henikoff *et al*. 2001; Malik 2009; Talbert & Henikoff 2022; Plačková *et al*. 2024). Through its effects on centromeric and kinetochore architecture, centromere drive may not only influence chromosome and genome size (Plačková *et al*. 2024) but also contribute to long‐term variation in recombination rates.

While our study focuses on a monocentric species, centromere/kinetochore architecture varies substantially across eukaryotes. In holocentric chromosomes, kinetochore activity is distributed along the chromosome length (Hofstatter *et al*. 2022), potentially altering the geometric and mechanical implementation of chromosome–kinetochore scaling. Moreover, even within monocentric systems, unusual architectures such as the macro‐monocentromeres described in *Chamaelirium luteum* (Kuo *et al*. 2025) or metapolycentric centromeres described in *Pisum sativum*, *Lathyrus sativus* (Neumann *et al*. 2012; Schubert *et al*. 2020; Macas *et al*. 2023), and in the beetle, *Tribolium castaneum* (Grzan *et al*. 2020) illustrate that centromere organization forms a continuum rather than a strict dichotomy. Comparative analyses, including holocentric and polycentric species, have demonstrated that the positive relationship between total centromere/kinetochore size and chromosome size is maintained across this architectural spectrum (Plačková *et al*. 2021). This suggests that scaling may represent a general organizational principle rather than an architecture‐specific feature. Further intra‐karyotype comparisons should help clarify how centromere and kinetochore structure shape scaling relationships.

Taken together, chromosome–kinetochore scaling can be viewed as the outcome of interacting short‐term cellular constraints and long‐term evolutionary processes that shape chromosome architecture while maintaining the functional requirements of chromosome segregation and recombination. The detection of this scaling relationship in Star of Bethlehem, despite relatively modest intra‐karyotype chromosome size variation, provides additional support for the consistency of this pattern in monocentric species and suggests that even subtle differences in kinetochore and centromere architecture may have far‐reaching consequences for genome evolution, including the evolution of recombination rates.

## Author Contributions

KP, MH and FZ designed the study. MH collected the plant material. KP performed lab work, image and statistical analyses. KP, MH and FZ wrote the manuscript.

## Supporting information


**Fig S1.** Example of measurement procedure in *O. kochii* (metaphase code NAA1_16s6). Raw images were analysed using ImageJ. KNL1‐labelled regions were determined from images enlarged to 400% using the ‘Elliptical selections’ function (right panel). Chromosome boundaries based on DAPI staining were defined from images enlarged to 300% using ‘Freehand selections’ tool (middle panel).

**Table S1.** List of localities of plants used in this study.
**Table S2.** Measurements of chromosome area and kinetochore area.

## Data Availability

All the data required to replicate the study are provided within the main body of the manuscript or Supporting Information – S1.
