# Supplementary figures and images for: Kinetochore size correlates with chromosome size in Star of Bethlehem (Ornithogalum kochii Parl., Asparagaceae)
Source: Plant Biol (Stuttg). 2026 Apr 14;28(5):1737–42. doi: 10.1111/plb.70215 (PMC13358698; doi:10.1111/plb.70215)

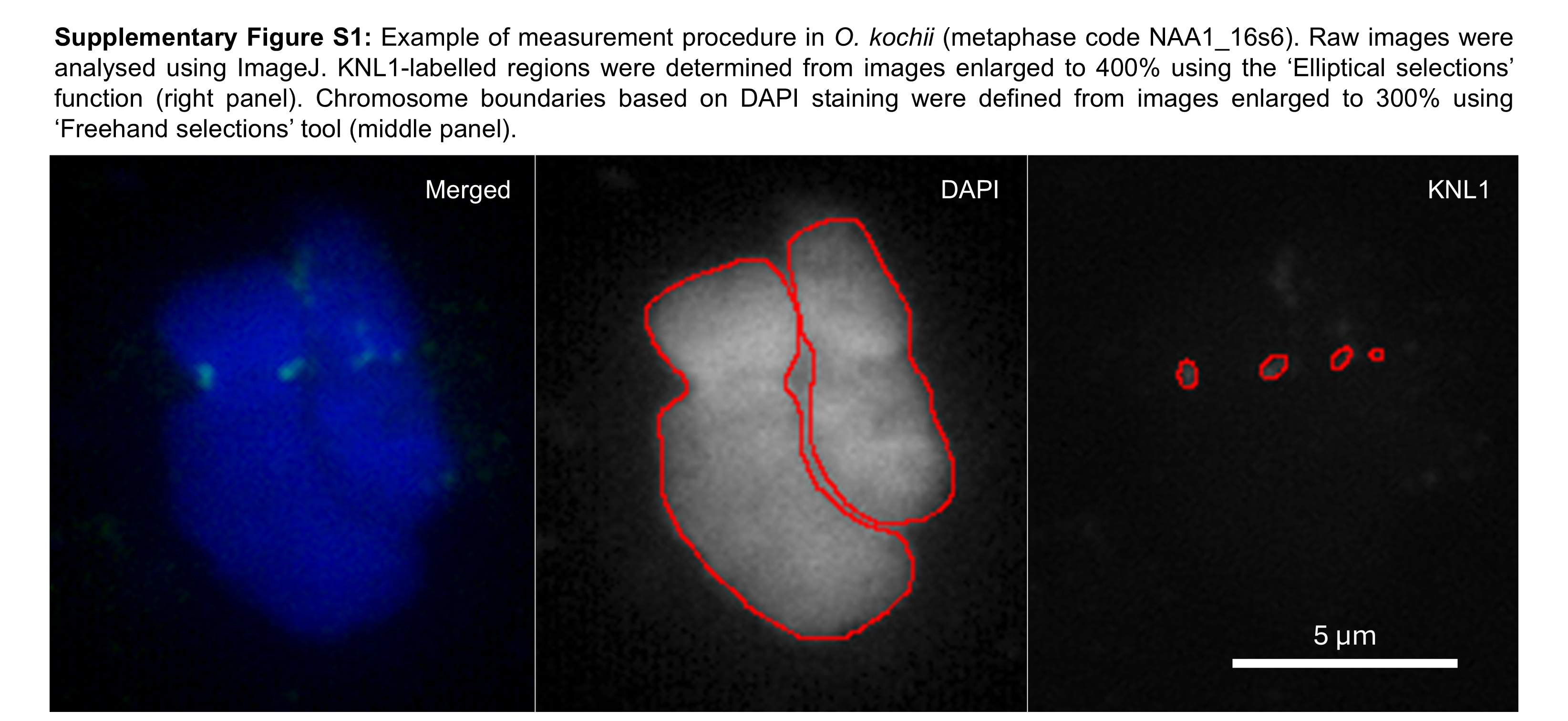

Supplement: Supplementary file 1 — Fig S1. Example of measurement procedure in O. kochii (metaphase code NAA1_16s6). Raw images were analysed using ImageJ. KNL1‐labelled regions were determined from images enlarged to 400% using the ‘Elliptical selections’ function (right panel). Chromosome boundaries based on DAPI staining were defined from images enlarged to 300% using ‘Freehand selections’ tool (middle panel). [file PLB-28-1737-s001.tif]
